# *In situ* metabolomics reveals intra-islet metabolite changes upon *in vivo* stimulation of insulin secretion

**DOI:** 10.1016/j.jbc.2025.110661

**Published:** 2025-09-01

**Authors:** Yan Zhou, Nicolas Baez, Tingting Fu, Thierry Brun, Pierre Maechler

**Affiliations:** 1 Department of Cell Physiology and Metabolism, University of Geneva Medical School, Geneva, Switzerland; 2 Department of Quantum Matter Physics, Laboratory of Advanced Technology, University of Geneva, Geneva, Switzerland

**Keywords:** pancreatic islet, beta-cell, metabolomics, insulin secretion, glutamate dehydrogenase

## Abstract

Upon glucose stimulation, the metabolic pathways of pancreatic beta-cells promptly adapt metabolite levels, inducing insulin secretion fine-tuned by mitochondrial glutamate dehydrogenase (GDH). Although well described *in vitro*, these responses cannot yet be captured *in vivo* due to the intrinsic nature of the islets scattered throughout the pancreas. Tested first *in vitro*, glutamate precursor glutamine enhanced glucose-stimulated insulin secretion without eliciting oxidative catabolism, as opposed to glucose. Then, to be as close as possible to the *in vivo* state, we collected the pancreas of mouse models in fasted *versus* fed states and at the peak of a glucose tolerance test, immediately followed by snap freezing before *in situ* analysis of metabolic pathways. On the same series of pancreatic cryosections, islets were identified by dithizone beta-cell staining for metabolic analyses combining spatial *in situ* redox enzymatic assay with targeted metabolomics using time-of-flight secondary ion MS high-resolution imaging. Direct measurements in cryopreserved pancreatic sections of control and beta-cell-specific GDH knockout mice showed tight coupling between glycolysis and mitochondrial pathways, favored by low lactate dehydrogenase activity and strong succinate dehydrogenase velocity. In response to regular feeding, intra-islet glutamate and glutamine levels were elevated, an effect dependent on beta-cell GDH. Acute *in vivo* glucose stimulation increased both alanine and glutamate intra-islet levels. Lack of beta-cell GDH abrogated the rise in glutamate and reduced insulin secretion without impacting alanine levels. Overall, the hallmark of *in vivo* beta-cell stimulation was a strong mitochondrial activity and GDH-dependent elevation of glutamate required for the full development of insulin secretion.

Pancreatic ß-cells are glucose sensors and produce corresponding amounts of insulin, then acting on target tissues to regulate glycemia. Upon glucose stimulation, activation of metabolic pathways in the ß-cell induces the elevation of cytosolic Ca^2+^ as the primary and necessary signal for insulin exocytosis. Then, increasing the magnitude of the secretory response requires amplification of the Ca^2+^ signal involving metabolism-derived additive factors (1, 2).

The process of metabolism-secretion coupling in ß-cells tightly relies on enzymes catalyzing redox reactions, essentially driven by dehydrogenases. Among them, glutamate dehydrogenase (GDH, encoded by *GLUD1*) is a mitochondrial homohexamer (3) catalyzing the reversible reaction α-ketoglutarate + NH_3_ + NADH ↔ glutamate + NAD^+^. GDH is at the crossroad of redox pathways, bridging carbohydrate and amino acid metabolisms through the breakdown or formation of glutamate that enters various metabolic pathways according to cellular demand and the specificity of tissue function (4). Different enzymes participate in the regulation of glutamate levels; *i.e*., GDH, alanine aminotransferase (ALAT), aspartate aminotransferase (ASAT), and glutaminase (5); but only GDH catalyzes deamination, resulting in net changes in the amino acid pool, potentially impacting the whole metabolome of the cell. GDH is allosterically modulated by leucine and adenine/guanine nucleotides (6, 7). In mammals, GDH is regulated negatively by GTP and positively by ADP (3), and inhibited by SIRT4 through reversible cysteine-specific ADP-ribosylation.

The importance of GDH as a key enzyme in the control of insulin secretion has been highlighted long ago (8). Its specific role in the amplifying pathway (9) points to glutamate enhancing the mandatory calcium signal by targeting the secretory granules (10), directly upon glucose stimulation (11) or favored by GLP-1 signaling (12). These complex mechanisms, not yet elucidated and not addressed in the present study, imply provision of glutamate upon glucose stimulation (9, 10, 11, 13, 14, 15). This has been shown *in vitro* including by tracer studies (9) but a putative corresponding effect remains to be investigated *in vivo*. Several research groups addressed the levels of metabolites in islets upon *in vitro* glucose stimulation, as extensively reviewed by Spégel and Mulder (16). Regarding glutamate, tracer studies using C-labeled glucose (12, 17, 18) and most (19, 20, 21, 22, 23), but not all (24), metabolomics studies based on mass spectrometry reported elevation of this amino acid upon *in vitro* glucose stimulation. Despite all these valuable investigations, we are still missing *in situ* delineation of the islet metabolism and GDH connected pathways under physiological *in vivo* conditions without *in vitro* stimulation. Upon an *in vivo* glucose challenge, metabolite levels within the islets remain unknown and cannot be assessed using NMR, as islets constitute less than 2% of the whole pancreas. As a proxy, in the present study we collected the pancreas of mouse models in the fasted state *versus* regular post-feeding, as well as at the peak of a glucose tolerance test, immediately followed by snap freezing before *in situ* analysis of metabolic pathways. This approach avoids islet isolation with potential metabolic resetting because of the culture procedure, as well as alternative preparations of tissue lysates and the loss of organ integrity. Practically, pancreas collection was performed in two conditions known to result in glucose-stimulated insulin secretion by the ß-cells, *i.e*., ad libitum feeding and upon acute *in vivo* glucose challenge.

The analyses conducted in this study consisted in the combination of spatial *in situ* redox enzymatic assay and metabolomics performed on the very same series of cryopreserved pancreatic sections, enabling the superimposition of these complementary layers of information. For the assessment of metabolites related to mitochondrial activation directly on the pancreatic sections, we used innovative time-of-flight secondary ion mass spectrometry (ToF-SIMS) for high resolution imaging at the cellular scale (25, 26). Collectively, the results show that *in vivo* stimulation of the ß-cell is associated with tight coupling of glycolysis with mitochondrial activation and a net elevation of glutamate levels in a GDH-dependent way.

## Results

### *In vitro*, glutamine potentiates glucose-stimulated insulin secretion without requiring its oxidation

**Figure 1:**
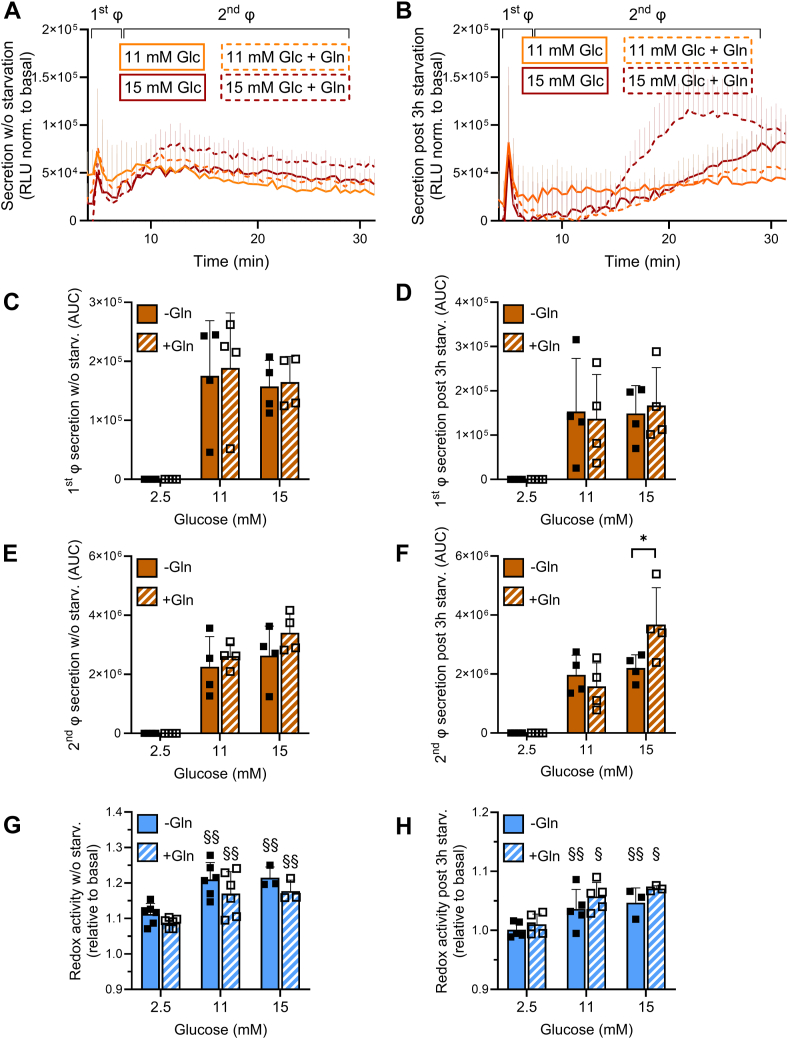
**Effects of glutamine on the kinetics of glucose-stimulated insulin secretion in INS-1E ß-cells.** After the standard culture period in regular RPMI-1640 medium (11.1 mM glucose), glucose-stimulated secretion was tested either (*A*) right away in the culture condition (w/o starvation) or (*B*) following a 3 h starvation period in glucose and glutamine-free medium. Secretion was monitored first for 5 min at basal 2.5 mM glucose and then at stimulatory 11 mM or 15 mM glucose (Glc) without or with 2 mM glutamine (Gln) for the remaining 30 min over the first and second phases (ϕ), n = 4. *C and D*, first ϕ secretion extracted from (*A*, *B*) and expressed as AUC for non-starved (*C*) or starved (*D*) INS-1E ß-cells. *E and F*, second ϕ secretion extracted from (*A*, *B*) and expressed as AUC for non-starved (*E*) or starved (*F*) INS-1E ß-cells. *G and H*, Redox activity assessed by the MTT assay performed in parallel and in the same conditions as the secretion assay on non-starved (*G*) or starved (*H*) INS-1E ß-cells. Values are means ± SD of independent experiments, using averaged triplicates in each independent experiment. Two-way ANOVA was used for multiple comparisons *versus* the basal 2.5 mM Glc group. ∗*p* < 0.05 *versus* corresponding w/o Gln group; ^§^*p* < 0.05, ^§§^*p* < 0.01 *versus* corresponding 2.5 mM Glc group.

Glucose is the primary stimulus of the pancreatic ß-cell, while other nutrients may modulate insulin secretion, such as fatty acids and amino acids (27, 28). Glutamine stimulates insulin secretion at basal glucose only when GDH is either experimentally robustly allosterically activated (27, 29, 30) or carrying pathological gain-of-function mutations (31, 32). However, exposure to glutamine rapidly raises intracellular levels of glutamate but without initiating insulin release on its own (33). *In vitro*, glucose-stimulated insulin secretion is typically measured in ß-cells following a preincubation period in nutrient-free medium, mimicking fasting by lowering intracellular metabolite stores and enabling a solid response to acute glucose stimulation. We first tested the contribution of glutamine (2 mM) in the secretory response to both intermediate and robust glucose stimulations (11 mM and 15 mM glucose, respectively; Fig. 1, *A* and *B*). In the absence of a starving period before the acute stimulation, glutamine did not change the secretory profile, neither over the first phase nor during the second phase. However, following a 3 h starvation period in glucose- and glutamine-free medium, the second phase of secretion evoked by 15 mM glucose was significantly increased by the presence of glutamine (+67%, *p* = 0.0215), while the first phase was not affected (Fig. 1, *C*–*F*). In parallel, we assessed the redox activity during these stimulation periods (Fig. 1, *G* and *H*), revealing that only glucose stimulation increased oxidative pathways. Upon glutamine exposure, redox activity was neither induced at basal glucose nor did it exert additive effects at stimulatory glucose concentrations. Of note, the potentiation of the secretory response by glutamine was absent with 1 or 2 h of starvation (data not shown). This set of data shows that in cells depleted of metabolite stores, glutamine amplifies the second phase of the glucose response without engaging its oxidative catabolism.

### Starvation impacts on islet mitochondrial activity as revealed by *in situ* measurements in cryopreserved pancreatic sections

*In vitro*, glutamine exposure sensitizes the ß-cells to acute glucose stimulation following starvation. Because glutamine is efficiently deamidated to glutamate, its fate as a potential substrate for GDH oxidative activity has been debated. The absence of GDH in ß-cells should tell, upon stimulation of insulin release, if GDH is required for potential changes in glutamate and glutamine levels. In order to be as close as possible to a physiological *in vivo* situation, we next collected the pancreas of control and ß-cell GDH knockout mice in various feeding conditions and immediately processed the organ in OCT medium by snap freezing in liquid nitrogen before analyzing.

**Figure 2:**
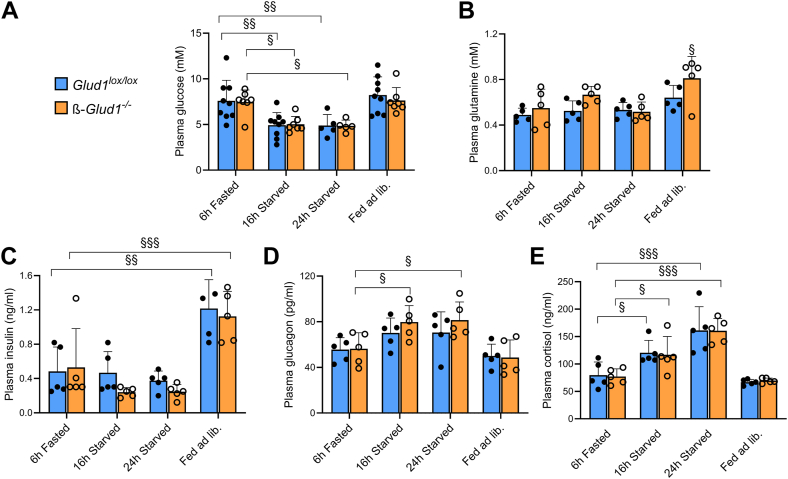
**Blood parameters of control *Glud1*^lox/lox^ and ß-cell GDH knockout ß-*Glud1*^−/−^ mice over various feeding conditions.** Mice were sacrificed and blood collected for plasma analysis after 6 h of fasting, 16 h or 24 h of starvation or fed ad libitum. *A*, blood glucose levels; plasma concentrations of (*B*) glutamine, (*C*) insulin, (*D*) glucagon, and (*E*) cortisol. Values are means ± SD, n = 5. Two-way ANOVA was used for multiple comparisons *versus* the 6 h fasted group; ^§^*p* < 0.05, ^§§^*p* < 0.01, ^§§§^*p* < 0.001; • *Glud1*^lox/lox^, ○ ß-*Glud1*^−/−^.

**Figure 3:**
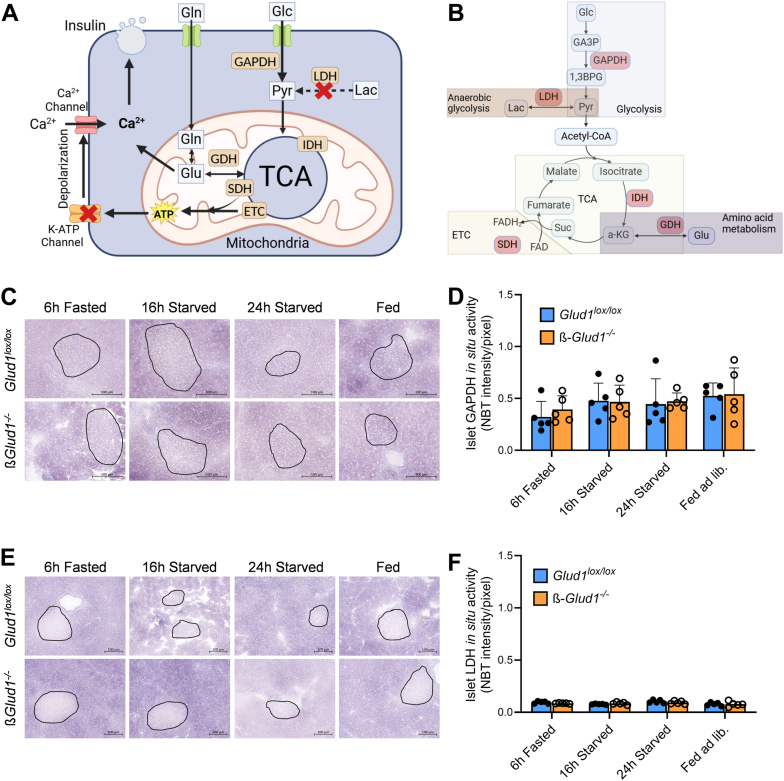
**Pancreatic *in situ* spatial assessment of islet glycolytic enzyme activities.** Mice were sacrificed after 6 h of fasting, 16 h or 24 h of starving, or fed ad libitum and pancreas were collected for *in situ* NBT enzymatic assay performed on pancreatic cryosections in control *Glud1*^lox/lox^ and ß-*Glud1*^−/−^ mice. *A*, simplified scheme of key metabolic pathways involved in metabolism-secretion coupling in ß-cells; glucose (Glc), pyruvate (Pyr), lactate (Lac), glutamine (Gln), glutamate (Glu), glyceraldehyde-3-phosphate dehydrogenase (GAPDH), lactate dehydrogenase (LDH), mitochondrial isocitrate dehydrogenase (IDH), succinate dehydrogenase (SDH), glutamate dehydrogenase (GDH), tricarboxylic acid cycle (TCA), electron transport chain (ETC). *B*, main metabolic pathways with their respective flagship enzymes; glycolysis (GAPDH), anaerobic glycolysis (LDH), TCA cycle (IDH), TCA-ETC coupling (SDH), amino acid bridge (GDH). *C*, representative *in situ* NBT enzymatic assay for GAPDH performed on pancreatic cryosections. *D*, quantification of islet *in situ* GAPDH activity. *E*, representative pancreatic *in situ* NBT enzymatic assay for LDH. *F*, quantification of islet *in situ* LDH activity. Islets are circled in black (based on DTZ staining), scale bar = 100 μm. Values are expressed as means ± SD, individual points correspond to distinct mice, n = 5. Two-way ANOVA was used for multiple comparisons; • *Glud1*^lox/lox^ mice, ○ ß-*Glud1*^−/−^ mice.

The blood parameters of *Glud1*^lox/lox^ and ß-*Glud1*^−/−^ mice are shown in Figure 2. As expected, glycemia lowered over prolonged starvation periods with corresponding changes in glucagon and cortisol levels. After ad libitum feeding, insulinemia increased in both *Glud1*^lox/lox^ and ß-*Glud1*^−/−^ mice, while plasma glutamine levels were slightly elevated in knockout mice. Pancreatic cryosections of the same mice were then examined for their main metabolic pathways assessed by the *in situ* measurements of corresponding enzymatic activities of flagship enzymes (Fig. 3, *A* and *B*). This was achieved by using the redox-sensitive NBT assay that allows spatial comparison of islet cells revealed by DTZ ß-cell staining *versus* adjacent acinar cells.

Assessed through glyceraldehyde-3-phosphate dehydrogenase (GAPDH) activity, the glycolytic capacity was comparable in endocrine *versus* exocrine cells (Fig. 3*C*). In the islets, glycolysis showed no significant differences between *Glud1*^lox/lox^ and ß-*Glud1*^−/−^ islets or among the various feeding conditions (Fig. 3*D*). However, exocrine zones exhibited moderate changes according to the feeding state with slight elevation of GAPDH activity after ad libitum feeding (Fig. S1*A*). Interestingly, anaerobic lactate dehydrogenase (LDH) activity was nearly absent in islet cells (Fig. 3*E*), while exhibiting robust activity in acinar cells (Fig. S1*B*). This is in agreement with the ß-cell concept of “disallowed” anaerobic pathway, lactate production from pyruvate being selectively repressed, thereby favoring mitochondrial funneling (34). The knockout of GDH in ß-cells did not result in the upregulation of LDH activity (Fig. 3*F*).

**Figure 4:**
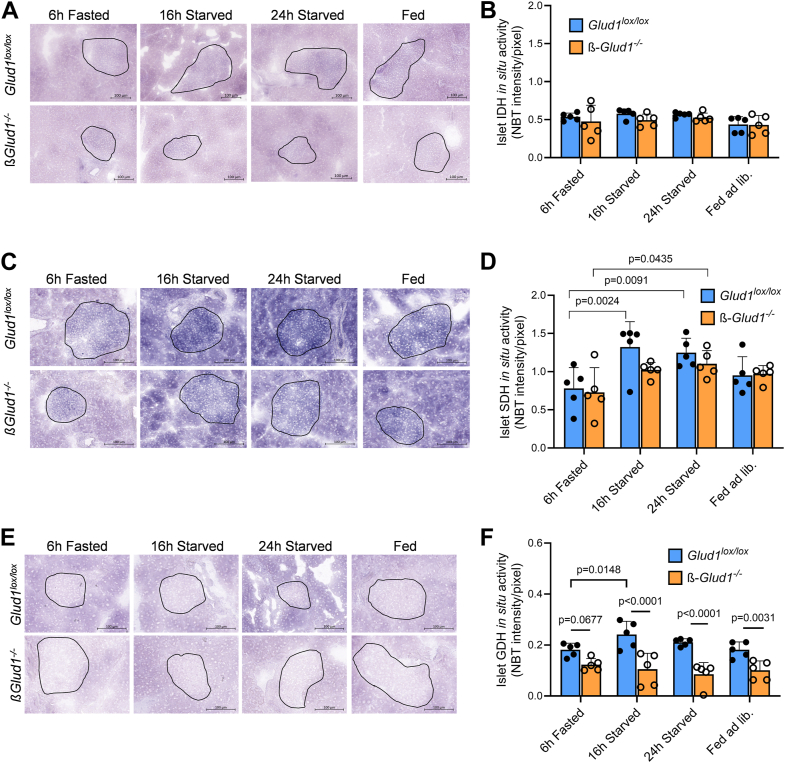
**Pancreatic *in situ* spatial assessment of islet mitochondrial enzyme activities.** Mice were sacrificed after 6 h of fasting, 16 h or 24 h of starving, or fed ad libitum and pancreas were collected for *in situ* NBT enzymatic assay performed on pancreatic cryosections in control *Glud1*^lox/lox^ and ß-*Glud1*^−/−^ mice. *A*, representative *in situ* NBT enzymatic assay for IDH performed on pancreatic cryosections. *B*, Quantification of islet *in situ* IDH activity. *C*, representative *in situ* NBT enzymatic assay for SDH performed on pancreatic cryosections. *D*, quantification of islet *in situ* SDH activity. *E*, representative pancreatic *in situ* NBT enzymatic assay for GDH. *F*, quantification of islet *in situ* GDH activity. Islets are circled in black (based on DTZ staining), scale bar = 100 μm. Values are expressed as means ± SD, individual points correspond to distinct mice, n = 5. Two-way ANOVA was used for multiple comparisons and corresponding actual *p* values are shown; • *Glud1*^lox/lox^ mice, ○ ß-*Glud1*^−/−^ mice.

Mitochondrial isocitrate dehydrogenase (IDH) activity was robust in islets, unaffected by feeding states or GDH expression (Fig. 4, *A* and *B*). As opposed to LDH activity, the enzyme succinate dehydrogenase (SDH), intersecting the TCA cycle and the electron transport chain, showed stronger velocity within islets compared to acinar zones (Fig. 4*C*), stressing the importance of the mitochondrial pathway for ß-cell function. Surprisingly, SDH enzymatic capacity was increased upon prolongation of the starving period, more rapidly in control islets compared to ß-cell GDH knockout islets (Fig. 4*D*). As expected, GDH activity was blunted in islets of ß-*Glud1*^−/−^ mice *versus Glud1*^lox/lox^ animals, while in the fasted state, its activity was close to background levels of the ß-cell knockout and lower than in acinar cells (Fig. 4, *E* and *F*). The full capacity of GDH can be prompted using BCH as a potent chemical allosteric activator (35). This confirmed more robust GDH activity in control islets under fed *versus* fasted states (Fig. S2). In acinar cells, GDH activity was slightly reduced in *Glud1*^lox/lox^ mice upon fasting, while IDH and SDH were not altered (Fig. S1, *C*–*E*).

This set of data shows, by direct measurements in the cryopreserved pancreatic sections, the strong coupling between glycolysis and the mitochondrial pathway favored by low LDH and strong SDH capacity.

### *In situ* metabolomics reveals GDH-dependent elevation of intra-islet glutamate levels in response to feeding

**Figure 5:**
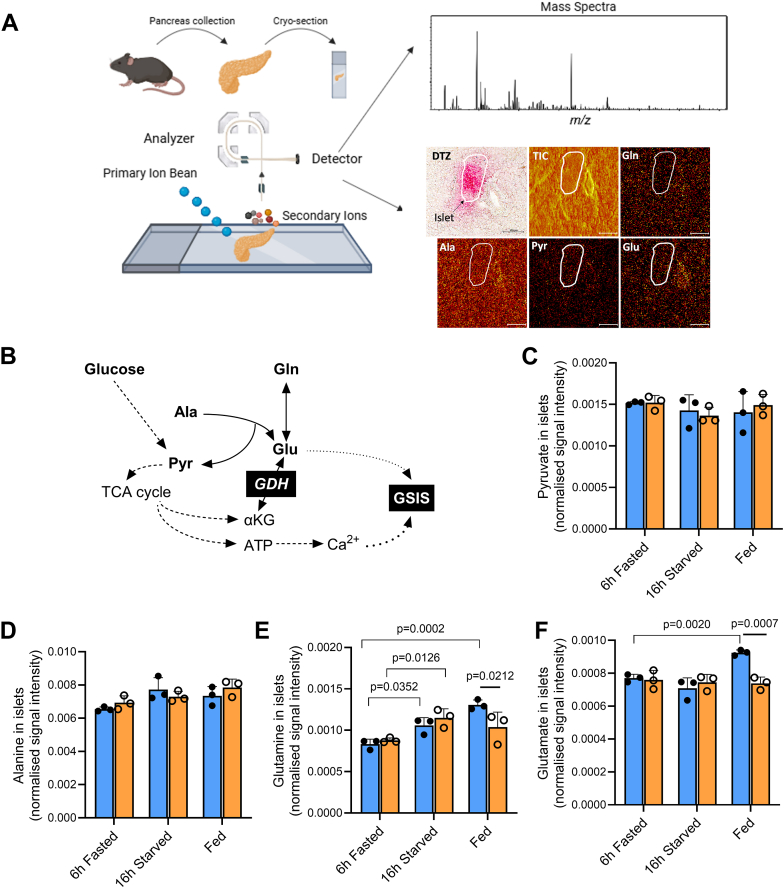
**Pancreatic *in situ* assessment of islet metabolites in fed and fasted states.** Mice were sacrificed after 6 h of fasting, 16 h of starvation, or fed ad libitum, and the pancreas were collected for *in situ* ToF-SIMS analysis on pancreatic cryosections in control *Glud1*^lox/lox^ and ß-*Glud1*^−/−^ mice. (*A*) Overview of *in situ* ToF-SIMS metabolomics workflow with islet circled according to DTZ staining and ion images of identified metabolites glutamine (Gln), alanine (Ala), pyruvate (Pyr), glutamate (Glu); TIC: Total Ion Counts; scale bar = 100 μm. *B*, metabolic pathways of the identified metabolites related to glucose-stimulated insulin secretion (GSIS). Based on the *m/z* ratio, normalized signal intensities in the islets were determined for (*C*) pyruvate, (*D*) alanine, (*E*) glutamine, and (*F*) glutamate. Values are means ± SD, individual points correspond to distinct mice, n = 3. Two-way ANOVA was used for multiple comparisons, and corresponding actual *p* values are shown; • *Glud1*^lox/lox^ mice, ○ ß-*Glud1*^−/−^ mice.

*In situ* ToF-SIMS metabolomics was conducted on pancreatic cryosections with DTZ staining on sequential slices to spatially identify the ß-cell area (Fig. 5*A*). ToF-SIMS allows semi-quantitative high spatial resolution assessment of the relative abundance of metabolites detected in the mass spectra (Figs. S3–S5). Of particular interest, pyruvate is the end product of glycolysis and a substrate for LDH unless efficiently metabolized by mitochondria. Pyruvate can also be formed by ALAT-mediated transamination of alanine; with transfer of its amino group to α-ketoglutarate, thereby producing glutamate (Fig. 5*B*). The levels of intra-islet pyruvate (C_3_H_3_O_3_^−^, *m/z* 87.008) and alanine (C_2_H_6_N^+^ fragment, *m/z* 44.050) were not changed by the feeding states and were similar between *Glud1*^lox/lox^ and ß-*Glud1*^−/−^ mice (Fig. 5, *C* and *D*). Compared to a 6 h fasting, both the post-feeding state and 16 h of starvation increased islet glutamine (fragments C_4_H_8_NO_2_^+^, *m/z* 84.044; C_4_H_11_N_2_O^+^, *m/z* 103.097) levels in control *Glud1*^lox/lox^ mice, while the elevation of islet glutamine after regular feeding was absent in ß-*Glud1*^−/−^ mice (Fig. 5E). Intra-islet glutamate (fragments C_4_H_6_NO_2_^−^, *m/z* 100.040; C_5_H_7_O_4_^−^, *m/z* 131.035) levels increased in response to ad libitum feeding in control mice, and this rise was totally abrogated in GDH knockout ß-*Glud1*^−/−^ mice (Fig. 5*F*).

These results show that intra-islet glutamine levels are increased upon conditions favoring glutamine availability, *i.e.,* through diet (post-feeding state) or gluconeogenic conditions (16 h starvation). Moreover, intra-islet glutamate levels are elevated specifically after feeding, an effect fully GDH-dependent.

### Intra-islet glutamate levels increase upon acute *in vivo* glucose stimulation

**Figure 6:**
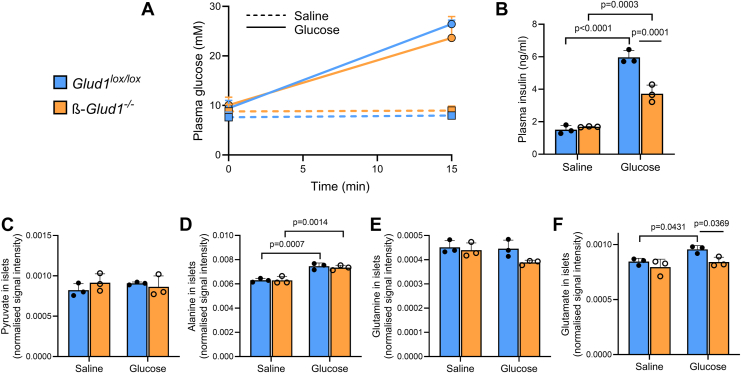
**Pancreatic *in situ* assessment of islet metabolites upon glucose tolerance test.** Partial i.p. GTT was performed on 4h fasted control *Glud1*^lox/lox^ and ß-*Glud1*^−/−^ mice injected with either 3 g/kg glucose (actual GTT) or saline (baseline glycemia). Animals were sacrificed at 15 min for pancreas collection. *A*, plasma glucose levels at times 0 and 15 min. *B*, plasma insulin concentrations at time 15 min. Based on the *m/z* ratio, normalized signal intensities in the islets were determined for (*C*) pyruvate, (*D*) alanine, (*E*) glutamine, and (*F*) glutamate. Values are means ± SD, individual points correspond to distinct mice, n = 3. Two-way ANOVA was used for multiple comparisons, and corresponding actual *p* values are shown; • *Glud1*^lox/lox^ mice, ○ ß-*Glud1*^−/−^ mice.

Even though glucose is the chief insulin secretagogue, the feeding state results in the elevation of a range of nutrients influencing the ß-cell response and islet metabolite load. Accordingly, we next performed *in vivo* acute stimulation just with glucose and measured intra-islet metabolites on cryopreserved pancreatic sections. Intraperitoneal GTT limited to time 0 (basal) and 15 min (acute ß-cell stimulation) showed the expected elevation of glycemia in both *Glud1*^lox/lox^ and ß-*Glud1*^−/−^ mice, while saline-injected mice remained at basal levels (Fig. 6*A*). Concomitantly, plasma insulin levels robustly increased in control mice, although the rise was blunted in GDH knockout ß-*Glud1*^−/−^ animals (Fig. 6*B*). This pattern is in agreement with previous results showing limited ß-cell response in ß-*Glud1*^−/−^ mice along with preserved glucose excursion secondary to increased insulin sensitivity (15). Beside the lack of ß-cell GDH, the latter effect might also, directly or indirectly, impact islet metabolism. ToF-SIMS analysis was performed on cryosections of pancreas collected at time 15 min post-injection of either glucose (GTT stimulation) or saline (basal state, time 0 equivalent). Intra-islet pyruvate levels were not changed upon *in vivo* glucose stimulation (Fig. 6*C*), indicative of a dynamic flux rather than an accumulation of this glycolysis-derived metabolite during metabolism-secretion coupling. Alanine is one of the metabolites that was consistently shown *in vitro* to be elevated in response to acute glucose stimulation (16). Here, *in situ* analysis confirmed this effect measured *ex vivo*, without differences between *Glud1*^lox/lox^ and ß-*Glud1*^−/−^ mice (Fig. 6*D*). As opposed to the ad libitum feeding response, *in vivo* glucose-restricted stimulation did not change intra-islet glutamine levels (Fig. 6*E*), suggesting an extra-islet exogenous source upon feeding. Finally, glutamate levels were increased upon acute *in vivo* glucose stimulation, an effect completely inhibited by the knockout of ß-cell GDH (Fig. 6*F*). The net elevation of amino acids might require the provision of ammonia, which should not be rate-limiting thanks to circulating NH_4_^+^ in the 22 to 72 μM range in mice (36).

Overall, *in situ* data show that acute *in vivo* glucose stimulation increases both alanine and glutamate intra-islet levels. Absence of ß-cell GDH specifically abrogated the rise in glutamate without impacting alanine levels.

## Discussion

Spatial *in situ* metabolomics using mass spectrometry imaging (MSI) is an emerging analytical strategy for in-depth investigation of tissue metabolic profiles (26, 37) based on label-free imaging and multiplexed mapping of molecules in a single biological tissue section (38, 39). By using a focused sampling probe (*e.g*. ion beam) generating charged species directly from the tissue pixel by pixel, MSI generates ion images providing the spatial distribution and relative abundance of molecules of interest. In this study, *in situ* metabolomics examined *in vivo* responses of individual intra-islet metabolites, providing complementary information (substrates and products) to the enzymatic assays (redox NBT) for a novel spatial delineation of intra-islet metabolic pathways.

Practically, ToF-SIMS imaging was performed on pancreatic cryosections targeting the regions of interest (islets) defined by DTZ ß-cell staining to provide unique metabolite mapping at the cellular scale, although it was not possible to discriminate subcellular compartments. Among the common molecular MSI techniques, ToF-SIMS could provide optimal spatial resolution and requires minimum sample preparation, avoiding the matrix peak interference issue commonly present in matrix-assisted laser desorption/ionization (MALDI) MS (40). Regarding limitations, quantification in MSI analysis remains challenging because of molecular ionization discrepancies caused by the inevitable matrix effect (25). Thus, absolute quantification of the metabolites was not pursued here. Nevertheless, since the chemical environment of a particular tissue structure (*e.g.*, islets in the whole pancreas) displays similarities between samples, one can evaluate the relative levels of individual metabolites by comparing the corresponding ion intensities. Here, the semi-quantitative assessment of the relative abundance of metabolites in the islets was carried out using normalized ion intensities.

Among the different metabolites of interest regarding metabolism-secretion coupling in the ß-cell, the role of glutamate remains debated as various actions mediating insulin release, targeting secretory granules or receptors, have been reported about this amino acid (10, 11, 12, 14, 41, 42). While the present study did not address these mechanisms, the net changes of its cellular levels upon glucose stimulation are discussed and dictate its action. Measured upon *in vitro* stimulation, most studies reported elevation of glutamate levels in islets upon glucose stimulation (16), an effect requiring the expression of GDH in ß-cells (9). Because GDH is under complex allosteric regulations, the balance of substrates and co-substrates on both sides of the reaction is not the only driver of flux direction. Mostly derived from liver-based studies, GDH properties (Km for substrates and co-substrates, mitochondrial pH) suggest that *in vivo* the reductive amination is favored (6, 43). For some authors the reaction is essentially the oxidative way (44), although this view is not shared by others (4, 45, 46, 47). Regarding the liver and the brain, our own data concur with a preferential oxidative GDH activity (48, 49, 50, 51). Conversely, in pancreatic ß-cells GDH might participate in glucose-stimulated insulin secretion through the formation of glutamate (9, 11, 15). Indeed, upon glucose stimulation, activation of the TCA cycle by the pyruvate dehydrogenase generates α-ketoglutarate and NADH, thereby promoting reducing activity of GDH (9, 11, 15). N-labelled tracer studies have shown that glutamine combined with the GDH allosteric activator BCH can compete with glucose flux in the TCA cycle (30). However, under physiological conditions GDH oxidative way to α-ketoglutarate is not favored in normal ß-cells, as demonstrated by the lack of secretory response triggered by glutamine (29, 52). Alternatively, glutamate could produce α-ketoglutarate by the actions of the aminotransferases ALAT and ASAT. However, such alternative pathways are at the expense of pyruvate and oxaloacetate, respectively, used as ammonia acceptor. Consequently, these reactions would consume intermediates of the metabolism-secretion coupling, unless they are operating in their corresponding shuttles, which are required for ß-cell function (53, 54). In this study, we did not observe changes in intra-islet pyruvate levels upon *in vivo* glucose-stimulated insulin secretion. However, both the post feeding state and an acute *in vivo* glucose challenge increased intra-islet glutamate levels, an effect fully GDH-dependent. Overall, the present study reports GDH-dependent elevation of islet glutamate levels under conditions avoiding *in vitro* stimulation, relying only on *in vivo* physiological responses. Nevertheless, the exact metabolic routes underlying such changes remain to be explored, as the GDH requirement might be indirect.

Because glutamine is made available by the skeletal muscles during starving periods for gluconeogenesis, it makes sense physiologically that ß-cell GDH prevents glutamine-induced insulin secretion to avoid hypoglycemia. Interestingly, we measured increased intra-islet glutamine levels in starved conditions. Glutamine can be efficiently deamidated to glutamate through glutaminase 2 (GLS2) expressed in ß-cells (55) and can potentiate, not initiate, the secretory response. However, its further oxidative pathway might not be operative under physiological conditions, unless GDH carries activating mutations causing undesired secretion and a hyperinsulinism syndrome resulting in severe hypoglycemia (31). At the mechanistic level, the reason why an anaplerotic oxidative catabolism of glutamate through GDH would not be efficient in pancreatic ß-cells during starving periods remains unclear. Further studies are needed to address this key aspect, which is highly relevant not only for our general understanding of the metabolism-secretion coupling but also for better management of the severe hyperinsulinism syndrome resulting from dysregulated GDH.

## Experimental procedures

### INS-1E ß-cell culture, luminescence-based secretion, and redox assay

Modified INS-1E ß-cells (RRID: CVCL_0351) expressing a *Gaussia* luciferase in place of the insulin c-peptide (56, 57) were used for luminescence-based secretion assay. Cells were cultured in RPMI-1640 GlutaMAX medium at 11.1 mM glucose supplemented with 10 mM HEPES, 5% (vol./vol.) heat-inactivated FCS, 100 U/ml penicillin, 100 μg/ml streptomycin, 1 mM sodium pyruvate, and 50 μM ß-mercaptoethanol. For secretion experiments, cells were starved for the indicated time (0–3 h) in glucose and glutamine-free RPMI-1640 medium, washed in KRBH before initiating the secretion assay by adding 5 μM of native coelenterazine (Nanolight Technologies, Pinetop, AZ). Then, cells were exposed to different concentrations (2.5 mM, 11 mM, 15 mM) of glucose with or without 2 mM glutamine for 40 min of luminescence recording using a Fluostar plate reader (BMG Labtech, Ortenberg, Germany) maintained at 37 °C (58). The data obtained from the plate reader were expressed as relative luminescence units (RLU).

Based on the same stimulation timeline as the secretion assay, the redox activity driven by dehydrogenases in stimulated INS-1E ß-cells was assessed using the MTT (3-(4,5-dimethylthiazol-2-yl)-2, 5,-5-diphenyl tetrazolium bromide) assay. The absorbance of the formazan was measured at 540 nm using the Fluostar plate reader as described previously (59).

### Animal models

*Glud1*^*fl/fl*^ floxed animals (*Glud1*^*tm1.1Pma*^, MGI:3835667) were crossed with mice expressing the *Cre* recombinase under the rat insulin promoter, resulting in ß-cell-specific GDH knockout ß-*Glud1*^*−/−*^ mice (15). As *Rip-Cre* mice, we used the *Tg(Ins2-cre)*^*23Herr*^ line (MGI:2387567) with no undesired brain (15) or hypothalamic recombination of the floxed gene (60, 61, 62). We used control and knockout mice of 12 to 16 weeks of age obtained from the same litters to optimize standardization of the mixed C57BL/6J × 129/Sv genetic background, avoiding inbred strain-specific phenotypes (63). As no sex differences were observed regarding islet metabolism of ß-*Glud1*^*−/−*^ mice (64), only male mice were used in this study. The mice were housed in our animal facility at the University of Geneva and maintained under a 12h light/dark cycle (light on at 7:00 AM) according to procedures approved by the animal care and experimentation authorities of the Canton of Geneva (GE5820A/CH32346). Mice were provided ad libitum access to the SAFE-150 diet (Safe).

### Tissue collections

On the day of sacrifice, *Glud1*^lox/lox^ and ß-*Glud1*^−/−^ mice were divided into four different feeding conditions: 6h fasted, 16h starved, 24h starved, and fed ad libitum (right after light on, up to 8:00 AM). Prior to sacrifice, glycemia was measured using an Accu Check Aviva glucometer (Roch Diagnostics). Blood was collected in sodium-heparin tubes (Sarstedt Inc) and centrifuged to obtain plasma. Pancreas were immediately collected and preserved in OCT (optimal cutting temperature) compound (CellPath) and stored at −80 °C for further cryostat sectioning.

For acute *in vivo* glucose stimulation, mice fasted for 4 h were injected intraperitoneally with either glucose (3 g/kg body weight, the actual GTT) or saline (for baseline glycemia). Glycemia was measured by glucometer at time 0 and 15 min post-injection. At 15 min, mice were sacrificed for blood and pancreas collections as described above before storage and further analysis.

### Plasma and pancreas analyses

The plasma concentrations of glutamine, insulin, glucagon, and cortisol were determined using commercial kits. Glutamine was measured by colorimetric assay (Abcam) and hormones by ELISA kits: insulin and glucagon (Crystal Chem); cortisol (Enzo, Farmingdale, NY).

OCT-preserved pancreatic cryosections of 8 μm thick were mounted on Super Frost PLUS slides (Menzel-Glaser). To visualize ß-cells in unfixed cryosections, which limits the accuracy of immunostaining, we used dithizone (diphenylthiocarbazone, DTZ) staining that reveals zinc-rich insulin granules (65). In brief, filtered DTZ (Sigma-Aldrich) solution (10 mg/ml) was applied onto cryosections, and the slides were incubated in a humid chamber at 37 °C for 30 min. Then, the cryosections were rinsed twice with PBS and once with Milli-Q water before being dried in a ventilation hood. The dried sections were mounted in glycerol and examined using a Zeiss Axio Scan Z1 slide scanner (Carl Zeiss Microimaging GmbH).

### *In situ* enzymatic activity

*In situ* enzymatic redox activity of the main targeted metabolic pathways was assessed on pancreatic cryosections by quantitative enzyme histo-biochemistry using the nitro blue tetrazolium (NBT) assay described previously (45, 51). Here, we measured the *in situ* enzymatic activities of glyceraldehyde-3-phosphate dehydrogenase (GAPDH), lactate dehydrogenase (LDH), isocitrate dehydrogenase (IDH), succinate dehydrogenase (SDH), and glutamate dehydrogenase (GDH) using the corresponding enzyme-specific substrates and cofactors. The specificity of the assay is primarily ensured by corresponding enzyme-specific substrate and co-substrate with validation based on kinetics and dose responses, like classic enzymatic assay on tissue extracts, which determined the conditions listed in Table 1. Practically, cryopreserved pancreas embedded in OCT were cut in cryostat to obtain 8 μm thick cryosections which were allowed to reach 37 °C in a dark humid chamber before adding reaction mix (see Table 1) in PBS buffer (0.1 M KH_2_PO_4_, 0.1 M Na_2_HPO_4_) with 18% of polyvinyl alcohol (PVA, Alfa Aesar), electron carriers phenazine methosulfate (PMS, Sigma-Aldrich) or 1-methoxy-PMS (mPMS, Interchim), specific substrates and cofactors (NAD^+^, Applichem; ADP, Sigma-Aldrich), and 5 mM NBT (prepared in 1:1 ethanol: dimethylformamide; Toronto Chemical). PVA was used to allow small molecules, such as substrates and cofactors, to freely diffuse in the reaction buffer, while proteins (such as target enzymes) in the tissue section are confined at the site of their *in situ* cellular localization. At the end of the incubation period (Table 1), the reaction was stopped by washing the slides twice with PBS at 60 °C for 10 min, followed by three washes with Milli-Q water. Then, slides were air-dried and mounted with glycerol for image acquisition.

**Table 1:** Composition of the enzymatic NBT assay according to the target enzyme

Enzyme	GAPDH	LDH	SDH	IDH	GDH
pH of PBS	7.4	7.4	8.0	7.4	8.0
NBT (mM)	5	5	5	5	5
NAD^+^ (mM)	1.5	1.5	0	1.5	1.5
ADP (mM)	0	0	0	0	1.0
NaN_3_ (mM)	5	5	0	0	0
PMS (mM)	0	0	0.32	0.32	0.32
mPMS (mM)	0.45	0.45	0	0	0
Substrate (mM)	glyceraldehyde-3-phosphate (2.5)	Lactate (10)	Succinate (0.6)	Isocitrate (2.0)	Glutamate (4.0)
Supplier	Cayman, Ann Arbor, MI	Combi-Blocks, San Diego, CA	Sigma-Aldrich, Burlington, MA	Sigma-Aldrich, Burlington, MA	Sigma-Aldrich, Burlington, MA
Incubation time (min)	30	10	30	30	30

Images were acquired under brightfield light on a Zeiss Axio Scan Z1 slide scanner using a 10x objective. The original brightfield images were converted to grayscale with black representing the lowest intensity level (value 0) and white the highest level (value 2). Grayscale images were analyzed using the Qupath software (Pete Bankhead) to quantify the NBT signal intensity per pixel. For each mouse, we measured the NBT signal converted to gray scale in islets revealed by DTZ ß-cell staining of each subsequent cryosection of the same OCT-preserved pancreas (Fig. S6). For each section, signal intensity was measured on five different DTZ-positive zones, which determined the average islet enzymatic activity per mouse. Measured in acinar cells, changes in enzymatic activity according to the feeding states, as well as GDH knockout, prevented normalization of endocrine *versus* exocrine zones (Fig. S1).

### High-resolution *in situ* metabolomics

*In situ* metabolomics was performed on pancreatic cryosections using high-resolution time-of-flight secondary ion MS (ToF-SIMS) imaging to allow discrimination at the cellular scale (26). For sample preparation, the frozen tissues embedded in OCT medium were sectioned at 8 μm thickness in a cryostat. Tissue sections were mounted onto stainless-steel slides and immediately transferred to −80 °C deep freezer. Before the analysis, the cryosections were brought to room temperature and dried in a desiccator under low vacuum for 30 min. To spatially navigate within the sections, ToF-SIMS was combined with DTZ staining performed on subsequent sections to identify islets as the regions of interest. ToF-SIMS imaging was performed on a PHI nanoToF II TOF-SIMS instrument (Physical Electronics) equipped with a 30 kV bismuth liquid metal ion gun (LMIG). The Bi_3_^2+^ primary ion beam was operated in bunched mode to achieve high mass resolution. With a sample bias of approximately 3 kV, the secondary ions were extracted into a triple focusing time-of-flight (TRIFT) analyzer consisting of three electrostatic analyzers and then detected by a dual microchannel plate (DMCP) detector. Regions of interest of 500 × 500 μm^2^ on the pancreatic sections were imaged with 256 × 256 pixels and a primary ion dose of approximately 2 × 10^12^ ions/cm^2^. Mass spectra were acquired over a mass range of *m/z* 0 to 1850 in both positive and negative polarities. Data processing was carried out using the PHI TOF-DR software (Physical Electronics). Internal calibration of the mass spectra was performed using small fragment peaks C_3_H_5_^+^, C_4_H_7_^+^, C_5_H_14_NO^+^, and C_5_H_15_NO_4_P^+^ in positive ion mode; and CH^-^, C_2_H^-^, CH_2_^-^, and C_4_H^-^ in negative ion mode. The ion peak assignment of the metabolites of interest, including both the pseudo molecular ions and the characteristic fragment ions, was achieved by referring to the ToF-SIMS spectra database for amino acids (66, 67). The positive ions at *m/z* 84.044 (C_4_H_8_NO_2_^+^) and *m/z* 103.097 (C_4_H_11_N_2_O^+^) were used to construct the ion image of glutamine and evaluate its abundance level (66). Fragment ion at *m/z* 44.050 (C_2_H_6_N^+^) was used to determine the alanine level (66). In negative ion mode, we selected ions at *m/z* 100.040 (C_4_H_6_NO_2_^-^), *m/z* 131.035 (C_5_H_7_O_4_^-^) to represent glutamate, and *m/z* 87.008 (C_3_H_3_O_3_^-^) for pyruvate (67). Regions of the islet were manually defined in the TOF-DR software by comparing ion images with DTZ staining on subsequent tissue sections. The metabolite ion intensities extracted from the islets were normalized to the regional total ion counts.

### Statistical analysis

The statistical analyses were performed by using GraphPad Prism 9.5 software (Graphpad Software Inc.). Differences between groups were analyzed by two-way ANOVA and were considered significant when *p* < 0.05. Values are expressed as means ± SD

## Data availability

All data generated during these studies are included in the text, figures, and tables of this article and the electronic supplementary material. Source data or materials will be supplied by the corresponding authors with reasonable request.

## Supporting information

This article contains supporting information.

## Conflict of interest

The authors declare that they have no conflicts of interest with the contents of this article.
