# Supplementary material for: In situ metabolomics reveals intra-islet metabolite changes upon in vivo stimulation of insulin secretion
Source: J Biol Chem. 2025 Sep 1;301(10):110661. doi: 10.1016/j.jbc.2025.110661 (PMC12493140; doi:10.1016/j.jbc.2025.110661)
Supplement: Supporting information [file mmc1.pdf]

Supplementary figures

Zhou et al.

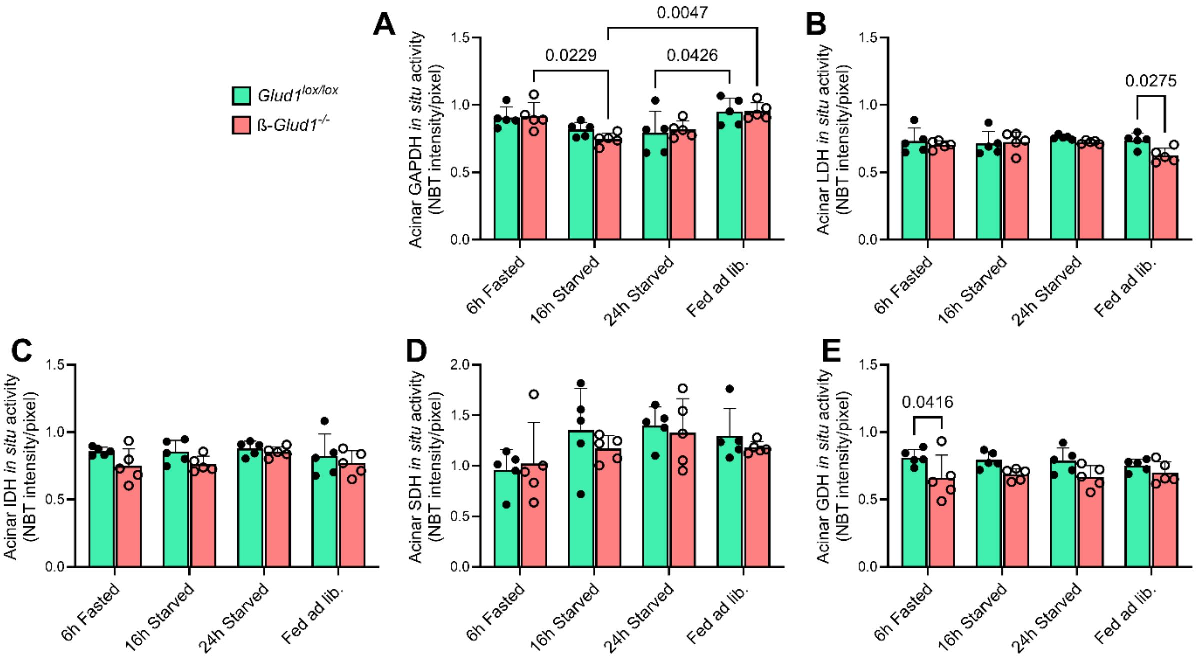

Figure S1: Pancreatic *in situ* spatial assessment of acinar enzyme activities. Mice were sacrificed after 6 h of fasting, 16 h or 24 h of starving, or fed ad libitum and pancreas were collected for *in situ* NBT enzymatic assay performed on pancreatic cryosections in control *Glud1*$^{lox/lox}$ and ß-*Glud1*$^{-/-}$ mice. Quantification of acinar activity of (**A**) glyceraldehyde-3-phosphate dehydrogenase (GAPDH), (**B**) lactate dehydrogenase (LDH), (**C**) isocitrate dehydrogenase (IDH), (**D**) succinate dehydrogenase (SDH), (**E**) glutamate dehydrogenase (GDH). Values are expressed as means ± SD, individual points correspond to distinct mice, n=5. Two-way ANOVA was used for multiple comparisons and corresponding actual p values are shown; ● *Glud1*$^{lox/lox}$ mice, ○ ß-*Glud1*$^{-/-}$ mice.

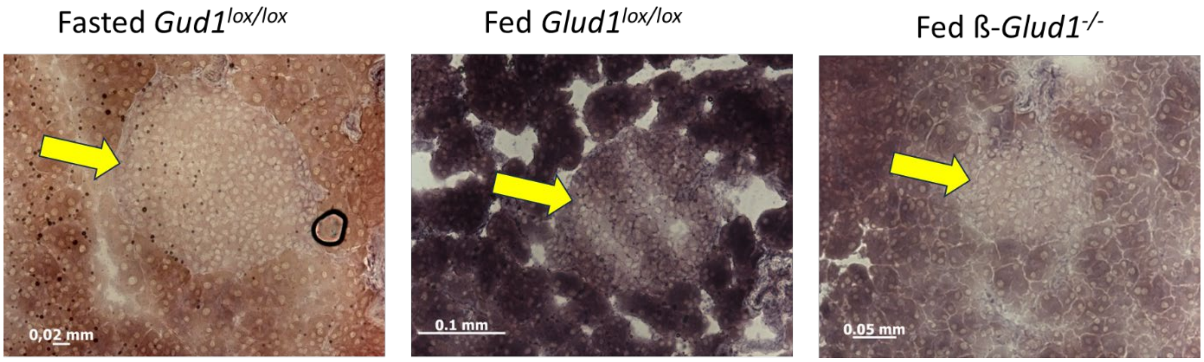

Figure S2: Pancreatic *in situ* GDH activity allosterically induced. Mice were sacrificed after 6h of fasting or after ad libitum feeding and pancreas were collected for *in situ* NBT enzymatic assay performed on pancreatic cryosections in control *Glud1*$^{lox/lox}$ and ß-*Glud1*$^{-/-}$ mice. Representative pancreatic *in situ* NBT enzymatic assay for GDH stimulated with 5mM glutamate and 10mM 2-aminobicyclo[2.2.1]heptane-2-carboxylic acid (BCH). Arrows indicate islets.

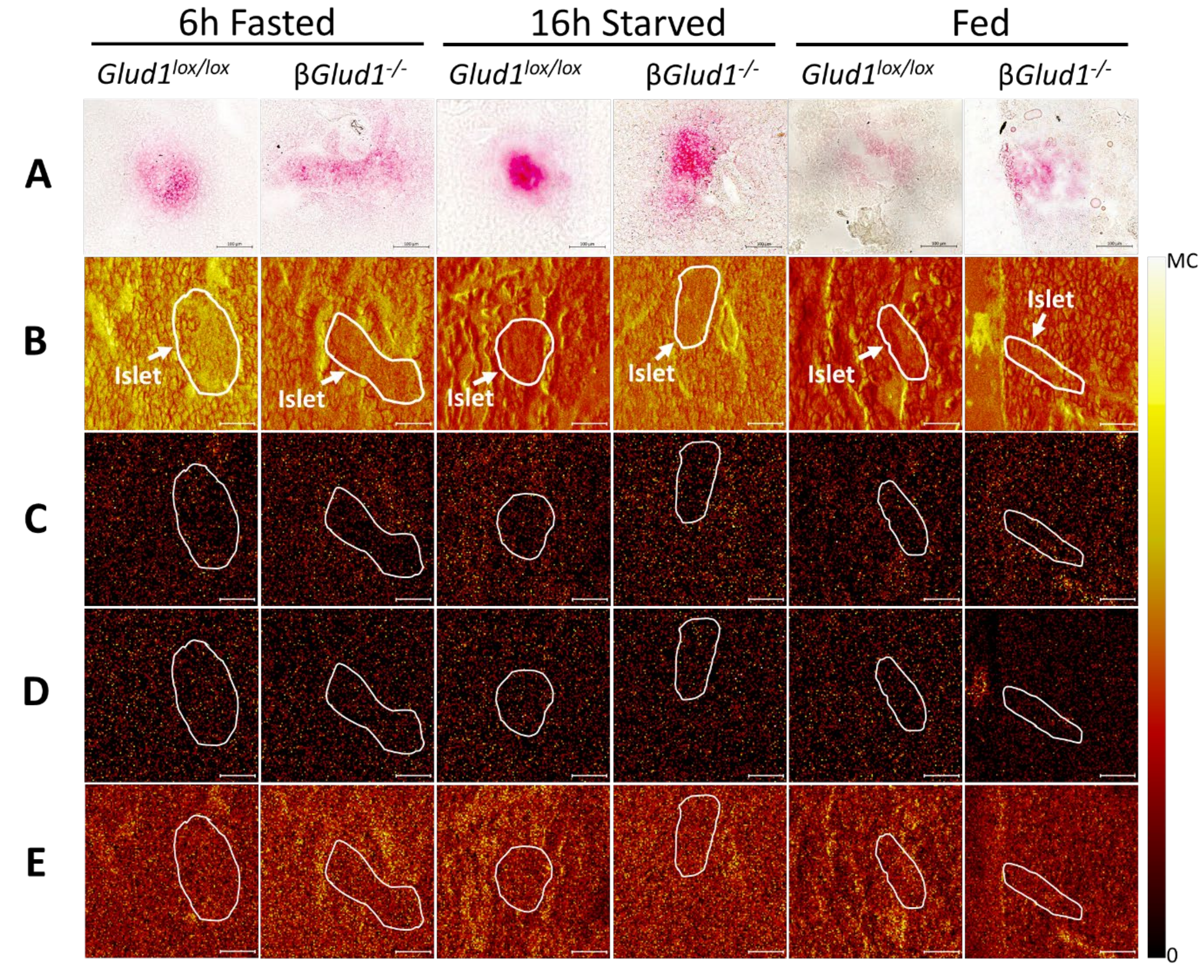

Figure S3: Representative ToF-SIMS positive ion images. (**A**) DTZ staining of pancreatic cryosections. On the subsequent slides (**B**) ToF-SIMS total ion count images of the analyzed pancreas cryosection with indicated islets and representative ToF-SIMS images of (**C**, **D**) glutamine-specific fragments (**C**, $C4H6NO^+$ *m/z*=84.044; **D**, $C4H11N2O^+$ *m/z*=103.097) and (**E**) alanine-specific fragment ($C2H6N^+$, *m/z*=44.050). Scale bar= 100µm. MC: maximum counts.

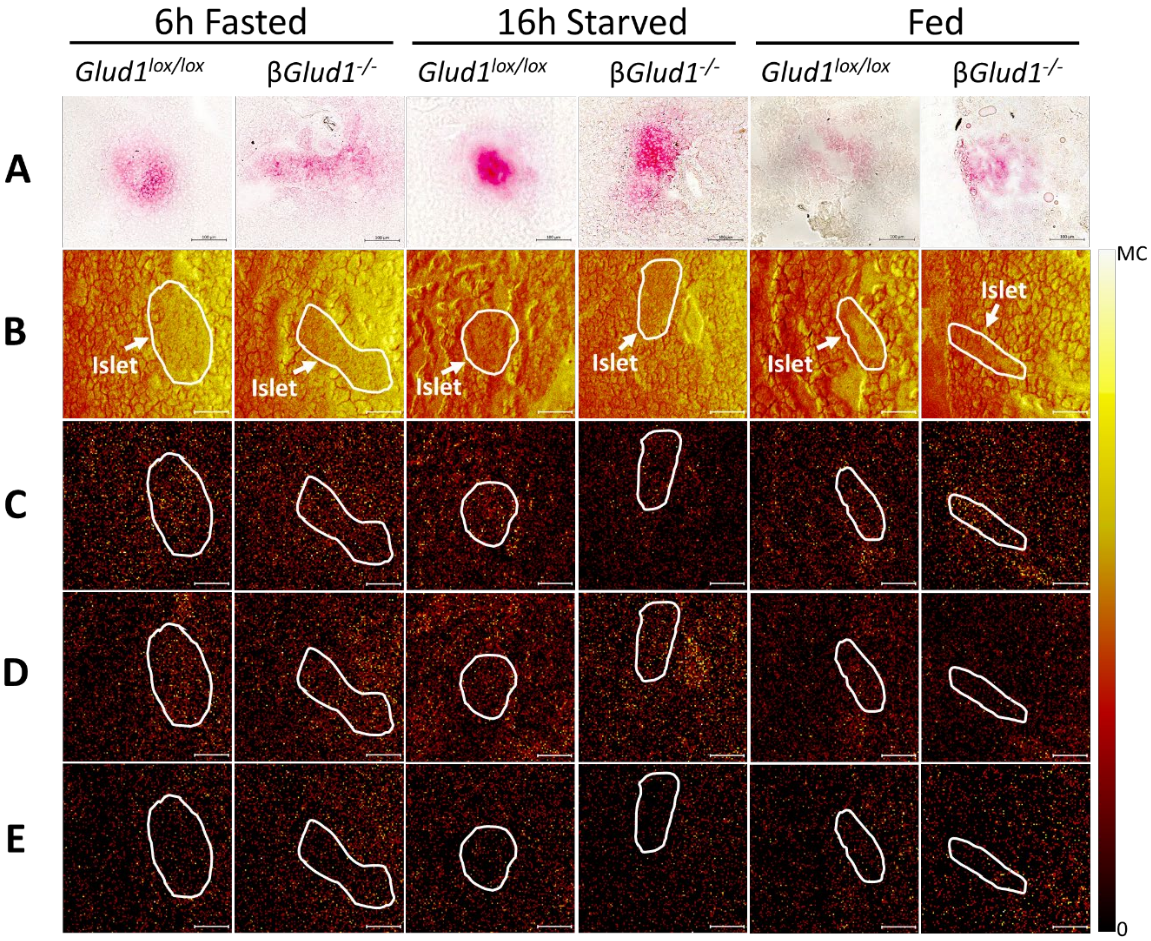

Figure S4: **Representative ToF-SIMS negative ion images**. (**A**) DTZ staining of pancreatic cryosections (same images as in Supplementary Figure S3A). On the subsequent slides (**B**) ToF-SIMS total ion count images (same as in Supplementary Figure S3B) of the analyzed pancreas cryosection with indicated islets and representative ToF-SIMS images of (**C**) pyruvate-specific fragment ($C3H3O3^{-}$, *m/z*=87.008) and (**D**, **E**) glutamate-specific fragments (**D**, $C4H6NO2^{-}$ *m/z*=100.040; **E**, $C5H7O4^{-}$ *m/z*=131.035). Scale bar= 100µm. MC: maximum counts.

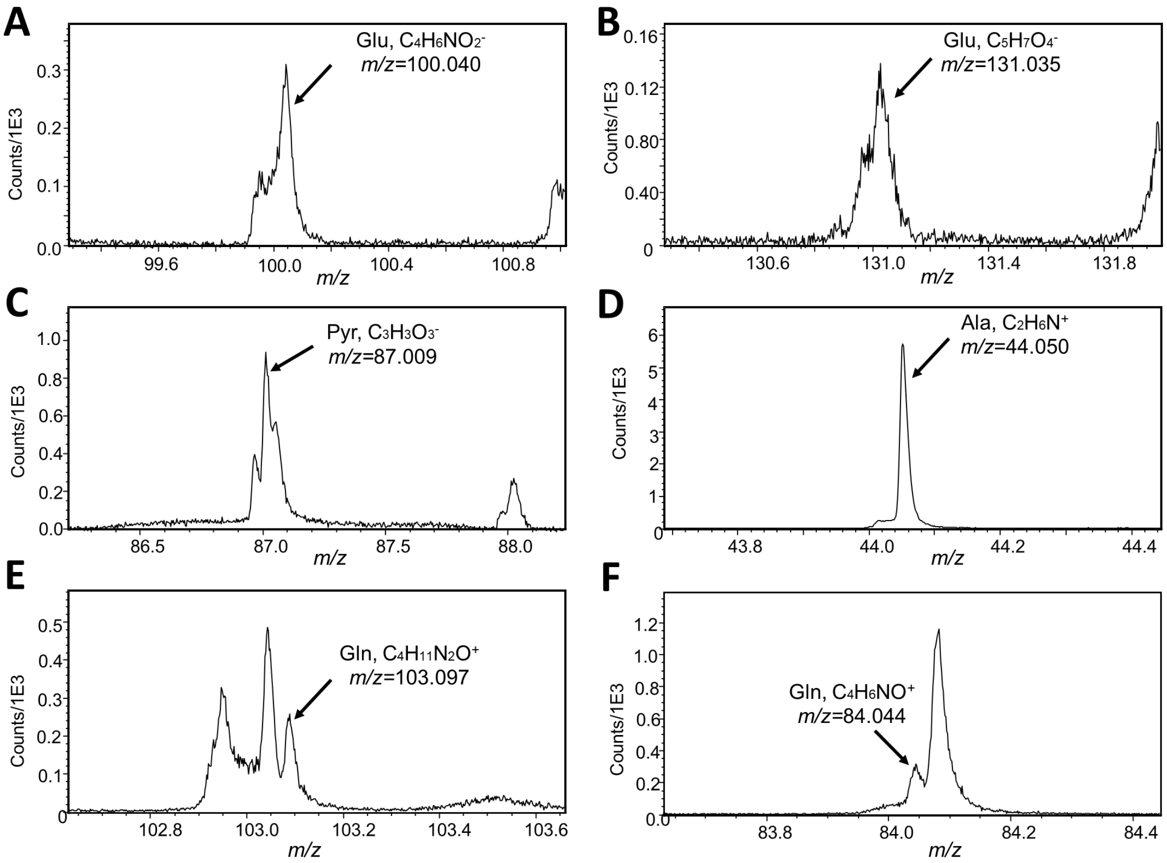

Figure S5: ToF-SIMS spectra of characteristic metabolite fragments in pancreas tissue of control *Glud1*$^{lox/lox}$ mouse sacrificed after 16h starving. (**A-C**) Negative ion ToF-SIMS spectra of glutamate (Glu) (*m/z*=100.040, $C4H6NO2^-$ and *m/z*=131.035, $C5H7O4^-$), and pyruvate (Pyr) (*m/z*=87.009, $C3H3O3^-$); (**D-F**) Positive ion ToF-SIMS spectra of alanine (Ala) ($C2H6N^+$, *m/z*=44.050), and glutamine (Gln) ($C4H11N2O^+$, *m/z*=103.097 and $C4H6NO^+$, *m/z*=84.044).

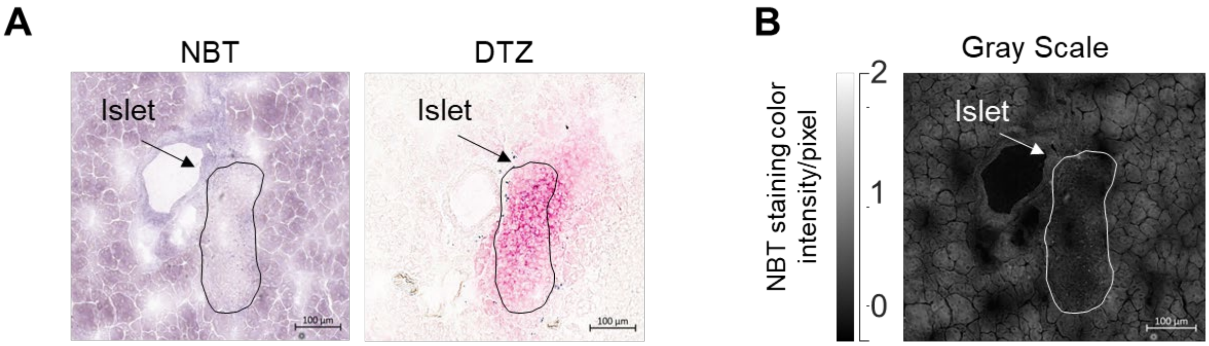

Figure S6: *In situ* navigation to identify islets in pancreatic sections. *In situ* NBT enzymatic assay performed on pancreatic cryosections in control mice. (**A**) Representative NBT assay showing both islet and acinar cell enzyme activities and, on subsequent pancreatic section (8 µm), DTZ staining revealing zinc-rich (insulin granules) ß-cells. (**B**) Quantification of NBT signal intensity by conversion of original staining into grayscale. Scale bar=100µm.
